# Autonomous beating rate adaptation in human stem cell-derived cardiomyocytes

**DOI:** 10.1038/ncomms10312

**Published:** 2016-01-19

**Authors:** George Eng, Benjamin W. Lee, Lev Protas, Mark Gagliardi, Kristy Brown, Robert S. Kass, Gordon Keller, Richard B. Robinson, Gordana Vunjak-Novakovic

**Affiliations:** 1 Department of Biomedical Engineering, Columbia University, New York, New York 10027, USA; 2 College of Physicians and Surgeons, Columbia University, New York, New York 10032, USA; 3 Department of Pharmacology, Columbia University, New York, New York 10032, USA; 4 McEwen Centre for Regenerative Medicine, University Health Network, Toronto, Ontario, Canada M5G1L7; 5 Department of Pathology and Cell Biology, Columbia University, New York, New York 10032, USA; 6 Department of Medicine, Columbia University, New York, New York 10032, USA

## Abstract

The therapeutic success of human stem cell-derived cardiomyocytes critically depends on their ability to respond to and integrate with the surrounding electromechanical environment. Currently, the immaturity of human cardiomyocytes derived from stem cells limits their utility for regenerative medicine and biological research. We hypothesize that biomimetic electrical signals regulate the intrinsic beating properties of cardiomyocytes. Here we show that electrical conditioning of human stem cell-derived cardiomyocytes in three-dimensional culture promotes cardiomyocyte maturation, alters their automaticity and enhances connexin expression. Cardiomyocytes adapt their autonomous beating rate to the frequency at which they were stimulated, an effect mediated by the emergence of a rapidly depolarizing cell population, and the expression of hERG. This rate-adaptive behaviour is long lasting and transferable to the surrounding cardiomyocytes. Thus, electrical conditioning may be used to promote cardiomyocyte maturation and establish their automaticity, with implications for cell-based reduction of arrhythmia during heart regeneration.

## Abstract (web summary)

The use of stem cell-derived cardiomyocytes for heart repair is hampered by their immature structural and contractile properties that may cause arrhythmia. Here, Eng *et al.* show that electrical conditioning of human cardiomyocytes in 3D culture can enhance connectivity and provide resistance to arrhythmia.

## Graphical abstract

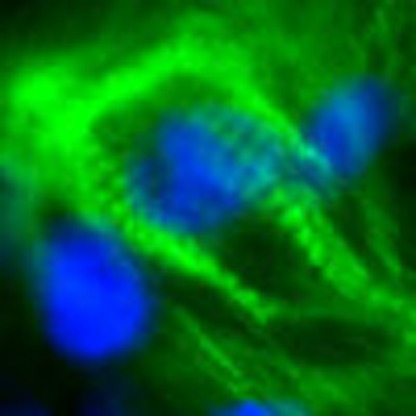

The burden of cardiovascular disease continues to grow, particularly due to the inability of the heart to repair itself after injury^1^,^2^. Procedures exist to derive cardiomyocytes from human embryonic and induced pluripotent stem cells^3^,^4^, and these cells provide unique potential to alleviate the burden of this epidemic^5^,^6^. While the delivery of cells to infarcted hearts has already begun^7^,^8^,^9^, the arrhythmogenicity of implanted cells pose a significant risk^10^. Two reasons are often cited, first related to the natural automaticity of nascent cardiomyocytes, where uncontrolled spontaneous beating can lead to ectopic foci of contraction^11^,^12^. Second, proper coupling via connexins is critical for the functional integration of cardiomyocytes to the host myocardium^13^,^14^. Therefore, techniques to control the beating rates and increase connexin expression of newly differentiated cardiomyocytes are becoming necessary to fully harness the therapeutic capacity of these cells.

A fundamental property of cardiomyocytes is their electromechanical excitability, where electrical depolarization triggers mechanical contraction and force generation^15^. Electrical signals, pervasive throughout life^16^,^17^ and critical to the cardiac environment^18^,^19^, are only beginning to be explored as a regulator of cell maturation and electromechanical function^19^,^20^,^21^,^22^,^23^,^24^,^25^. We hypothesize that electrical stimulation can structurally mature human stem cell-derived cardiomyocytes and alter their intrinsic beating properties.

To this end, nascent cardiomyocytes are cultured as three-dimensional embryoid bodies (EBs) formed from human embryonic or induced pluripotent stem cells (hESCs or iPSCs) using a staged molecular differentiation (Fig. 1a; Supplementary Fig. 1)^26^,^27^. Electrical signals are delivered continuously for 7 days using a custom-designed microbioreactor capable of providing multiple stimulation regimes (Fig. 1b). Three stimulation frequencies are chosen: 0.5, 1 or 2 Hz, with an unstimulated control (Fig. 1b). We show that electrical stimulation matures cardiomyocytes by enhancing connexin expression and sarcomeric structure. Uniquely, cardiomyocytes respond to electrical signals by adapting their autonomous beating rate to the rate at which they are stimulated. This adaptive effect is mediated in part by the enrichment of a rapidly depolarizing cell type, and by human ether-à-go-go-related gene (hERG), a voltage-gated potassium channel responsible for repolarization. Blockade of hERG abrogates the rate adaptation. The resultant cardiomyocytes are robust, maintain the adapted beating rates for up to 2 weeks and transfer this property to surrounding cells.

## Results

### Electrical stimulation matures cardiomyocytes

**Figure 1:**
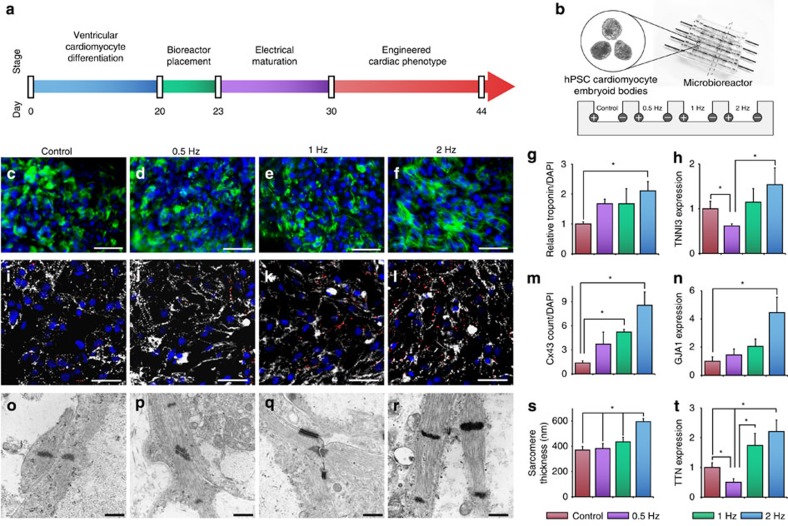
Electrical stimulation matures stem cell-derived cardiomyocytes. (**a**) Staged differentiation protocol for generating cardiomyocytes from hESCs or iPSCs. Cells were differentiated for 20 days, electrically stimulated for 7 days and taken off stimulation for 14 days to examine the lasting effects of electrical stimulation. (**b**) Schematic of microbioreactor set-up. Differentiated hESC- or iPSC-derived cardiomyocytes were placed into a polydimethylsiloxane bioreactor between parallel carbon rods with stimulation groups: unstimulated, 0.5, 1 and 2 Hz. (**c**–**g**) Immunostains demonstrating increasing levels of troponin (green) and improved organization with increasing frequency of stimulation. Slides were counterstained with 4,6-diamidino-2-phenylindole (DAPI, blue). Scale bar, 50 μm; n≥3. (**h**) Quantitative PCR of TNNI3 shown as a fold change relative to the control (average ±s.e.m., n≥3). (**i**–**m**) Immunostains demonstrating increasing levels of connexin-43 (red) with increasing rate of stimulation. Slides were counterstained with α-actinin (grey) and DAPI (blue). Scale bar, 25 μm; n≥3. (**n**). Quantitative PCR of GJA1 (average±s.e.m. of fold change relative to control, n≥3). (**o**–**r**) Transmission electron microscopy at × 20,000. Scale bar, 500 nm. (**s**) Sarcomere thickness (nm±s.e.m; *n*=8–14). (**t**) Quantitative PCR of TTN shown as a fold change relative to control±s.e.m. *Statistically significant differences between the individual groups or between multiple groups (*n*≥3, *P*<0.05, one-way analysis of variance (ANOVA) with *post hoc* Tukey tests).

At the end of just 1 week of electrical frequency stimulation, cardiomyocytes subjected to 2-Hz signals underwent hypertrophy (Supplementary Fig. 2) and had a more developed contractile apparatus compared with control, as evidenced by aligned striations and a greater expression of troponin (Fig. 1c–h; Supplementary Fig. 3a). Cells stimulated at 2 Hz also expressed a greater number of connexin-43 gap junctions, suggesting the formation of electrically coupled multicellular units (Fig. 1i–n; Supplementary Fig. 3b).

On the ultrastructural level, sarcomeres in unstimulated cells were thin and disorganized, oriented in multiple directions and only occasionally associated with Z lines. In contrast, stimulated groups contained myofibrils that were aligned in parallel across multiple cells, with the 2-Hz group showing remarkably well-developed striated ultrastructure with long continuous repeats of thick sarcomeric subunits (Fig. 1o–t).

Genes encoding for markers of cardiac differentiation (*Nkx2.5*), cardiac chamber specification (*NPPA*, *MYL2* and *ANKRD1*), structural development (*MYH6* and *IGF1R*) and electrical development (*KCNJ2*) were all upregulated with stimulation (Supplementary Fig. 4). There was no significant enrichment in the number of cardiomyocytes (Supplementary Fig. 1d), suggesting that the increased gene expression was due to changes in cardiomyocytes rather than in their relative fraction within the population of cells assessed. Together, these data demonstrate that electrical stimulation, especially at 2 Hz, matures the structure of newly differentiated cardiomyocytes and facilitates their assembly into contractile units.

### Cardiomyocytes adapt to the electrical stimulation frequency

**Figure 2:**
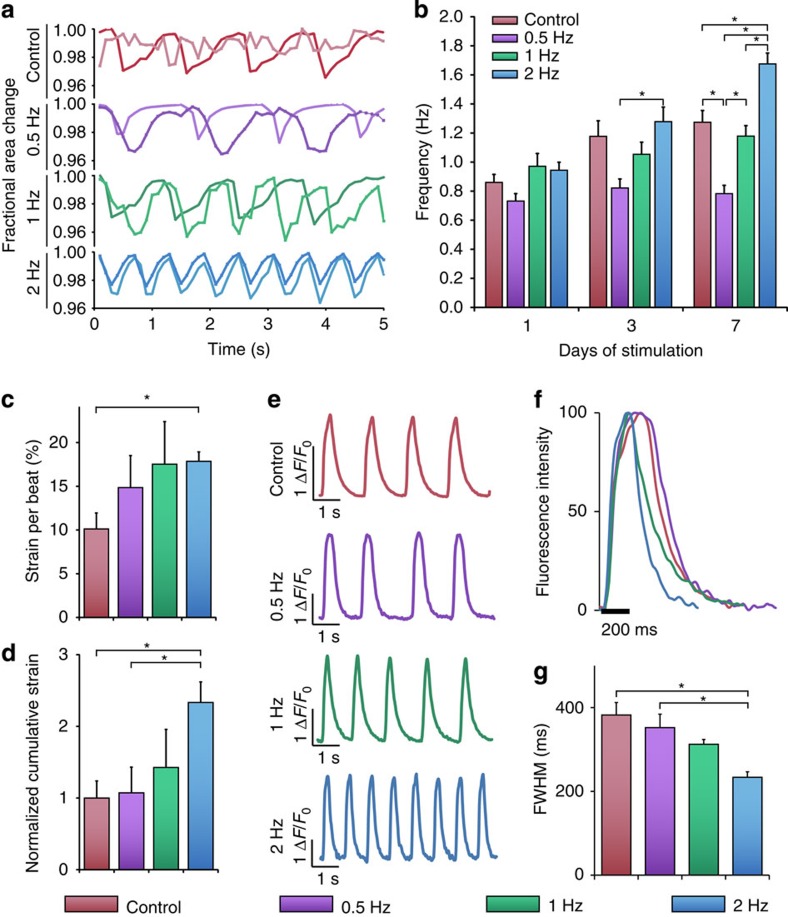
Electrical stimulation regulates automaticity in human stem cell-derived cardiomyocytes. (**a**) Fractional area change as a function of time after 7 days of stimulation. Dark and light traces represent two independently beating areas within the same field of view. (**b**) Frequency of autonomously beating cardiomyocytes as a function of duration of stimulation (average±s.e.m., *n*=30, *P*<0.05). (**c**,**d**) Strain rate per beat and the cumulative contraction strain (average±s.e.m., *n*=3, *P*<0.05). (**e**) Calcium fluorescence traces for an entire beating area over time. (**f**) Normalized fluorescence intensity over one beat. (**g**) Full width half maximum (FWHM) calcium transients for stimulated groups (average±s.e.m., *n*=23, *P*<0.05). *Statistically significant differences between groups, testing was done using one-way ANOVA with *post hoc* Tukey tests.

To examine the effect of electrical stimulation frequency on the contractile behaviour of stem cell-derived cardiomyocytes, we measured the spontaneous beating rate, a fundamental property of the cardiomyocyte^15^, at the end of the stimulation period. Most interestingly, electrical stimulation conditioned the automaticity of the cardiomyocytes, such that even after the removal from stimulation, the spontaneous beating rate remained adapted to the frequency of the electrical stimulus (Fig. 2a,b; Supplementary Movies 1–4; Supplementary Figs 3c and 5). Each group had unique responses to electrical stimulation. Specifically, the beating rate of cells in the 2-Hz-conditioning group was overdriven by and adapted to the electrical signal, showing an inducible increase in the automaticity (Fig. 2b; Supplementary Fig. 3c). Cells stimulated at 1 Hz maintained an ∼1 Hz spontaneous beating rate, similar to the natural rate of the control unstimulated cells. Cells stimulated at 0.5 Hz had a significantly lower spontaneous beating rate, but did not match the 0.5-Hz stimulus, evidenced by incomplete capture of the 0.5-Hz stimulus and the preservation of more rapidly beating overdriving cells innate to the cardiomyocyte population.

To determine whether the changes in the beating rates seen with electrical stimulation resulted in modifications of contractile function and electromechanical coupling, we conducted strain mapping and calcium imaging on spontaneously beating cells at the end of the 7-day stimulation period. Strain maps were generated by tracking pixel movements in a frame-to-frame cross-correlation algorithm (Supplementary Fig. 6). Average strain per contraction over the entire contractile area was the highest for the 2-Hz-conditioned group. This group also exhibited the highest overall cumulative strain, calculated as the amount of contractile strain generated in a given time (Fig. 2c,d). These data suggest that electrical stimulation improved contractile function in at least two ways as folllows: (i) by increasing the strain per contraction and (ii) by increasing cumulative strain, a measurement coupling the strain per contraction with the frequency of contraction. Electromechanical coupling, measured via high-speed video acquisition of cardiomyocytes stained with a calcium-sensitive dye Fluo-4 (Supplementary Fig. 7a) displayed similar frequency-dependent changes (Fig. 2e–g; Supplementary Fig. 7b,c). All measured transients—time to peak, full width half maximum and the time constant of decay (tau)—were accelerated by electrical stimulation, indicating enhanced calcium cycling (Fig. 2g; Supplementary Fig. 7b,c). The corresponding increases in the expression of calcium handling genes ryanodine receptor 2 (*RYR2*), sodium–calcium exchanger (*SLC8A1*) and L-type calcium channel (*CACNA1*) in the stimulated groups are consistent with maturation of the electromechanical coupling machinery (Supplementary Fig. 7d).

### *KCNH2* mediates the adaptation of beating rate

Taken together, these data suggest that the nascent human cardiomyocytes are responsive to electrical signalling, which can set their autonomous beating rate to the frequency of stimulation and enhance their electromechanical function. To identify potential mediators of beating rate adaptation, we conducted gene expression profiling (Supplementary Fig. 8) without additional purification of the cardiomyocytes. Overall, broad expression differences were found after 1 week of electrical stimulation, with 106 upregulated genes and 143 downregulated genes. Specifically, markers of non-cardiac differentiation, such as those for vascular endothelium (*VWF*) and non-cardiac muscle (*CDH6*, *CNN2* and *MYLK*) were downregulated, while genes encoding for cardiac hypertrophy (*IGF1R*) and calcium handling (*CAMK2B*) were upregulated. Among the most upregulated genes were *KCNH2*, encoding for hERG, the potassium channel responsible for repolarization of the cardiomyocytes, and *GJA5*, encoding for connexin-40 (Cx40), a gap junction channel responsible for rapid conduction between cells.

**Figure 3:**
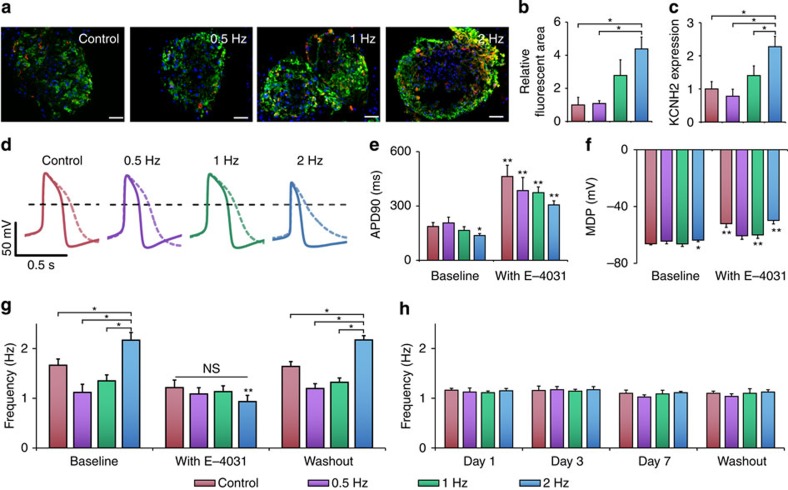
Conditioning of the beating frequency by electrical stimulation is mediated by *KCNH2*. (**a**,**b**) Immunostains of troponin (green) and hERG (red), counterstained with 4,6-diamidino-2-phenylindole (DAPI, blue); scale bar, 50 μm. Frequency-dependent increases in Troponin and hERG (*n*=3, *P*<0.05, one-way ANOVA with *post hoc* Tukey test). (**c**) qPCR of *KCNH2* confirms the upregulation with stimulation (average±s.e.m. of fold change). *Statistically significant differences between the 2-Hz group and the 1-, 0.5-Hz and control groups (*n*=3, *P*<0.05, one-way ANOVA with *post hoc* Tukey test). (**d**) Representative action potentials at baseline (solid) and after addition of the *KCNH2* inhibitor E-4031 (dashed). The dashed black line represents 0 mV. (**e**,**f**) Action potential duration (APD90) and maximum diastolic potential at baseline and when exposed to E-4031. *Significant difference between experimental electrically stimulated groups and control unstimulated cells at baseline. **Significant difference for an experimental group before and after treatment with E-4031 (control: *n*=13, 0.5 Hz: *n*=5, 1 Hz: *n*=9, 2 Hz: *n*=15, *P*<0.05, two-way ANOVA with *post hoc* Tukey test). (**g**) The differential beating frequency after 7 days of stimulation was abolished with the addition of the E-4031 and was regained after washing out the drug. *Statistically significant differences between the 2-Hz group and the 1-, 0.5-Hz and control groups (*n*=8, *P*<0.05). NS denotes no statistical difference between control, 0.5-, 1- and 2-Hz groups when exposed to E-4031. **Statistically significant difference for the 2-Hz group between the baseline, when exposed to E-4031, and following the washout of E-4031 (*n*=8, *P*<0.05, two-way ANOVA with *post hoc* Tukey test). (**h**) Frequency of cardiomyocytes throughout the stimulation period in the presence of E-4031, and following the washout of E-4031 after the stimulation period (*n*=8).

Differential expression of *KCNH2* suggested a mechanism through which hESC- or iPSC-derived cardiomyocytes respond to electrical conditioning. The expression profiling was validated using quantitative PCR (qPCR) that showed increased expression of *KCNH2* with increasing frequency of electrical stimulation (Fig. 3c; Supplementary Fig. 3b). Immunofluorescent staining further confirmed increased expression of hERG in the higher frequency stimulated groups on a protein level (Fig. 3a,b). The control, 0.5- and 1-Hz groups demonstrated sparse and focal staining, while the 2-Hz group had large regions that were positive for both hERG and troponin (Fig. 3a).

On the basis of its increased expression in stimulated groups, we investigated the role of hERG in modifying the action potentials across the groups (Fig. 3d–f; Supplementary Figs 9 and 10). Cells were patch clamped as small clusters of cells, where spontaneous beating rates in the 2-Hz group remained faster than the other three groups despite cellular dissociation (Supplementary Fig. 10a). When the activity of hERG was inhibited using a specific small molecule antagonist E-4031 at the end of the stimulation period, the repolarization phase of the action potential was prolonged (Fig. 3d; Supplementary Fig. 9), resulting in a twofold prolongation of the action potential (Fig. 3e).

In addition, inhibition with E-4031 increased the maximum diastolic potential (MDP) across the groups, with the 2-Hz group showing the greatest increase to the highest MDP of −49.8 mV (Fig. 3f). This behaviour is consistent with the reliance of the nascent cardiomyocytes on *I*_Kr_, where channel antagonism significantly increases their MDP^28^. Interestingly, there was no significant change in the rate of phase 4 depolarization (Supplementary Fig. 10b), which is often cited to dictate the spontaneous rate of cells^29^,^30^,^31^. Therefore, increased *KCNH2* in the 2-Hz-stimulated group appears to modify the spontaneous beating frequency of cardiomyocytes through a shortened action potential and an increased MDP.

We investigated the role of *KCNH2* in the rate-adaptive effect resulting from electrical stimulation. The application of E-4031 at the end of the stimulation period abolished differences in spontaneous beating rate induced by electrical stimulation (Fig. 3g). The greatest effect was observed in the 2-Hz group, where the average beating rate decreased by 57%, as compared with the decreases of 16% and 3%, respectively, in the 1 and 0.5-Hz stimulation groups. These changes were consistent with the corresponding changes in the expression of *KCNH2*, where overexpression of *KCNH2* caused the cells to be increasingly sensitive to E-4031. Subsequent removal of E-4031 permitted complete rescue of the differential automaticity.

To determine whether *KCNH2* function was necessary to establish beating rate adaptation, E-4031 was supplemented to culture media during the electrical conditioning period (Fig. 3h). In the presence of E-4031, the cells never developed frequency-dependent differences in their spontaneous beating rate, and instead maintained their frequency close to the natural beating rate of 1 Hz. Interestingly, even after removing E-4031 following the 7-day conditioning period, differential beating frequencies were not observed, suggesting that blocking the *KCNH2* inhibits the ability for cells to adapt to electrical stimulation frequency. These data implicate *KCNH2* as an essential factor for establishing and maintaining the adapted beating rates in nascent cardiomyocytes.

### Electrical stimulation enhances connexin expression

We also studied the effects of electrical stimulation on expression of connexins typically found in the heart. *GJA1* (Cx43), the primary gap junction in working cardiomyocytes and *GJA5* (Cx40), the rapidly conducting gap junctions typically found in secondary ventricular conduction cells (His Bundle, Purkinje fibres), were upregulated (Fig. 1i–n; Supplementary Fig. 11a,b), whereas *GJC1* (Cx45) and *GJD3* (Cx31.9), slowly conducting gap junctions typically found in nodal cells^32^, trended downward (without statistical significance; Supplementary Fig. 11a).

### Stimulation-dependent emergence of rapidly depolarizing cell

**Figure 4:**
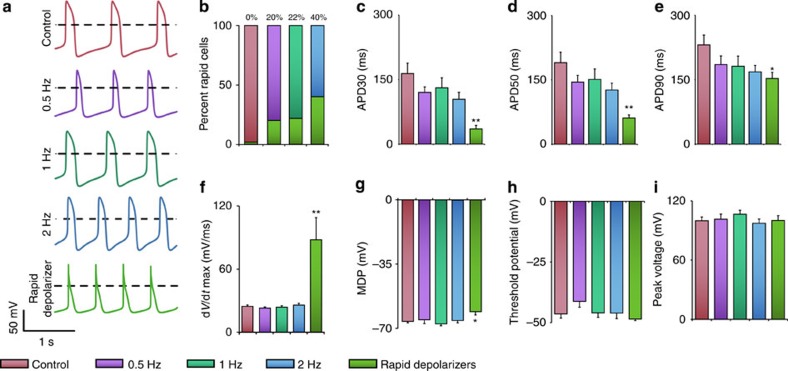
Electrical conditioning results in a subpopulation of rapidly depolarizing cells. (**a**) Representative action potential traces of the unstimulated, 0.5-, 1- and 2-Hz-stimulated groups. A subset of cells within the stimulated groups displayed unique rapid depolarizing action potentials; the dashed black line represents 0 mV. (**b**) The percentage of rapid depolarizing cells increased with increasing stimulation frequency, with no observed rapid depolarizing cells in the control group. (**c**–**e**) Action potential durations 30, 50 and 90 (APD30, APD50 and APD90) were significantly shorter in the rapid depolarizer group. (**f**) Rapid depolarizers in the 2-Hz group had characteristic high rates of depolarization, with d*V* d*t*_max_^−1^ approximately three times higher than in ventricular-like cardiomyocytes. (**g**) Maximum diastolic potential was significantly less negative in the 2 Hz rapid depolarizer group than any other group. (**h**) Peak voltage and (**i**) threshold potential were similar across all stimulation regimes, as well as in the rapid depolarizing cells. *Significant difference between electrically stimulated groups and the unstimulated baseline, **Significant difference between rapid depolarizing group and control, 0.5 Hz, 1 Hz, and 2 Hz groups. (control: *n*=13; 0.5 Hz: *n*=4; 1 Hz: *n*=7; 2 Hz: *n*=9; rapid depolarizer: *n*=9; *P*<0.05, one-way ANOVA with *post hoc* Tukey test).

Patch-clamp measurements revealed a subset of cardiomyocytes that had an action potential characterized by a steep depolarizing slope, distinct from the ventricular-like action potential morphology that contained a plateau phase (Fig. 4)^4^. Forty per cent of cardiomyocytes in the 2-Hz group exhibited this phenotype, and importantly, this phenotype was not observed in the control group, suggesting that electrical stimulation was necessary for generating the rapidly depolarizing cells (Fig. 4b). This emergent cell type had significantly shorter action potentials and a greatly increased d*V* d*t*_max_^−1^=88 mV ms^−1^, that was threefold higher than the average of d*V* d*t*_max_^−1^=24 mV ms^−1^ in all other cell groups (Fig. 4c–f)^33^. The rapid depolarization may be partially explained by the increased expression of sodium channel *SCN5A* (Supplementary Fig. 12). Together with the upregulation of the connexin-40-positive cells, this may suggest that electrical stimulation enriches for more rapidly conducting cells, in line with the previous observations from developmental studies^34^. These connexion-rich, rapidly depolarizing cells, may therefore allow for rapid communication between cardiomyocytes and the rate adaptation to the frequency of stimulation.

### Robust long-lasting effects of electrical stimulation

**Figure 5:**
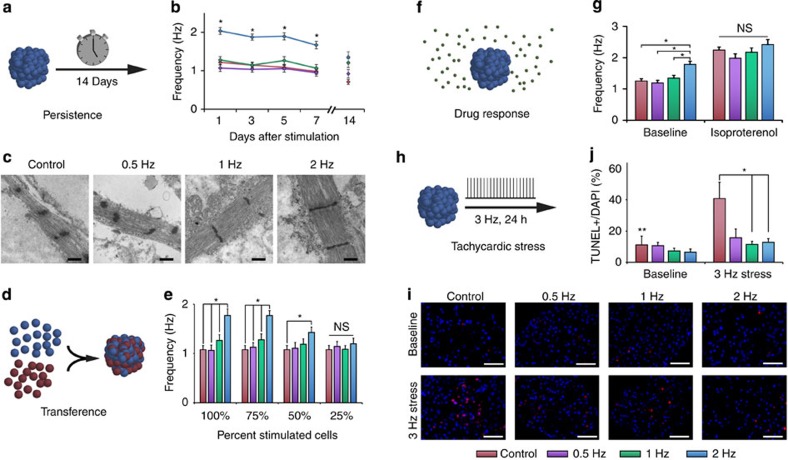
Robustness of electrical stimulation. (**a**,**b**) Maintenance of differential frequency for up to 2 weeks after stimulation. *Statistically significant differences between the 2-Hz group and the control group (*n*=10, *P*<0.05, one-way ANOVA with *post hoc* Tukey test). (**c**) Representative transmission electron microscopy of cardiomyocytes that were unstimulated, or stimulated at 0.5, 1 and 2 Hz after 7 days of stimulation and an additional 7 days after the stimulation was discontinued. Scale bar, 500 nm. (**d**,**e**) Transference of rate in electrically stimulated cardiomyocytes that were mixed at different ratios with unstimulated cells. Chimeric embryoid bodies containing 2-Hz-stimulated and unstimulated cells beat significantly faster than control samples (up to 50%). Displayed as average±s.e.m. *Statistically significant differences between the 2-Hz group and the 1-, 0.5-Hz or control groups (*n*=20, *P*<0.05, one-way ANOVA with *post hoc* Tukey test). (**f**,**g**). All groups displayed physiologic increase in frequency in the presence of 1 μM isoproterenol and maintained frequency conditioning trends. Displayed as average±s.e.m. *Statistically significant differences between the 2-Hz group and the 1-, 0.5-Hz and control groups. NS denotes nonsignificance between groups when stimulated with isoproterenol. (*n*=10, *P*<0.05, one-way ANOVA with *post hoc* Tukey test) (**h**–**j**) At baseline, all cells demonstrated low levels of TUNEL positivity (red). After being subjected to a frequency of 3 Hz for 24 h, control cells demonstrated the highest level of apoptosis, while the cells stimulated at 2 Hz showed low levels of apoptosis. Blue: 4,6-diamidino-2-phenylindole (DAPI). Scale bar, 50 μm. Apoptosis quantification displayed as average±s.e.m. *Statistically significant differences between the control group and the 1- and 2-Hz-stimulated groups. **Statistically significant difference between baseline and 3-Hz-stimulated control groups (*n*=3, *P*<0.05, two-way ANOVA with *post hoc* Tukey test).

Given the broad effects of electrical stimulation on the electrical, mechanical and developmental properties of nascent cardiomyocytes, we next investigated how these cells maintain their contractile behaviour after discontinuing electrical stimulation. Remarkably, the ability of the cells to adapt their automaticity to the rate of stimulation persisted for 2 weeks after the electrical stimulation was discontinued (Fig. 5a,b; Supplementary Fig. 3d). Even after the beating rates were no longer significantly different (at 2 weeks after the electrical stimulation was removed), the ultrastructure still revealed frequency-dependent changes, with the 2-Hz-stimulated group showing the widest sarcomeres and the control group showing thin and underdeveloped sarcomeres (Fig. 5c).

We next tested whether stimulated cells were able to affect the rate of surrounding cardiomyocytes by forming new chimeric EBs from various ratios of stimulated and unstimulated cells (Supplementary Fig. 13). The newly formed clusters contained a single focus of beating as observed by bright-field videos after 3 days of aggregation. At fractions of ≥50% of 2-Hz-stimulated cells, the entire chimeric EB was beating significantly faster than control cells, implying that stimulated cells may transfer the rate-adaptive effect to surrounding unstimulated cells via entrainment (Fig. 5d,e)^35^. Importantly, while electrical conditioning altered the cell automaticity, the cells were responsive to chronotropic drugs, demonstrating a physiologic increase in beating frequency in the presence of adrenergic stimulus isoproterenol (Fig. 5f,g).

Finally, we tested the ability of stimulated cells to tolerate rapid electrical pacing in a model of tachycardia established *in vitro*, which is known to lead to apoptosis of cells^36^,^37^,^38^. We stimulated the frequency-conditioned cells at 3 Hz for 24 h and observed the extent of apoptosis by terminal deoxynucleotidyl transferase dUTP nick end labelling (TUNEL) staining. Cardiomyocytes conditioned by electrical stimulation were largely unaffected by increased field stimulation frequency, while the stimulus naive control cells experienced a 30% increase in apoptosis (Fig. 5h–j). These results suggest that frequency-conditioned cells may be more resistant to further electrical insults. Therefore, this stimulation-induced robustness was associated with (i) maintenance of the acquired beating frequency after the stimulation was discontinued, (ii) transfer of this property to the surrounding cells, (iii) physiological responsiveness to chronotropic drugs and (iv) resistance to electrical pacing stress.

## Discussion

We demonstrate functional benefits of electrical stimulation for human cardiomyocytes derived from hESCs and iPSCs. Cell culture in three-dimensional aggregates rather than as single cells allowed the study of properties emergent from a population of cells, including multicellular architecture, beating frequency and transfer of the rate-adaptive effect. These phenomena are dependent not only on the cell–cell interactions but also on spatial cues from the local microenvironmental niche^35^,^39^. Tissue function becomes especially important for modelling of disease^40^, reliable drug testing^41^ and therapeutic applications of cells^42^.

Electrical stimulation matured cardiomyocytes, promoted the formation of multicellular contractile units and enhanced their electromechanical function. This is in line with other studies, where electrical stimulation, alone or in combination with mechanical loading can mature cardiomyocytes^22^,^25^,^43^. The degree of maturation of cardiomyocytes that would be necessary for therapeutic use, such as injection into the infarcted myocardium, remains an open question^5^,^44^. Some groups have shown functional benefits of released factors from nascent cardiomyocytes or even undifferentiated iPSCs in the *in vivo* setting^45^,^46^. Others have shown utility of vascularized tissue-engineered constructs^9^,^47^ used for remuscularizing the heart.

Uniquely, we show that electrical stimulation frequency alone can tune the ultimate maturity level of cardiomyocytes, thus allowing controlled studies on the effect of maturity on ultimate success as a therapy. We therefore envision that electrical stimulation can become a preconditioning method, where cardiomyocytes are matured to a specified developmental stage before introduction into injured myocardium.

We demonstrate that cardiomyocytes entrain to the frequency at which they were stimulated. This represents the first demonstration that cardiomyocyte beating frequency can be programmed. The 2-Hz stimulation group entrained to the 2-Hz stimulus, and beat at this rate for over 1 week following the cessation of stimulation. The spontaneous beating rate of the 1-Hz-stimulated cardiomyocytes was similar to the control cardiomyocytes. Surprisingly, despite the presence of overdriving cells, the 0.5-Hz-stimulated cells had a lower beating rate than the control cells. This demonstrates that the presence of electrical stimulation, independent of electromechanical capture, can still alter cardiomyocytes, consistent with previous work with electrical pacing in the presence of electromechanical uncoupler 2,3-butadione monoxime^24^. Like other groups, we found that automaticity was not mediated by hyperpolarization-activated cyclic nucleotide-gated channels, responsible for phase 4 depolarization and *I*_f_ (refs 15, 39). Consistent with these prior studies, we see that the maintenance of MDP is important to the ultimate spontaneous excitability of cardiomyocytes^15^,^39^. Interestingly, we show that rate adaptation was mediated at least in part by hERG, which has recently been shown to set the MDP in stem cell-derived cardiomyocytes^28^.

Our studies with electrical stimulation and hERG necessitate further exploration of the regulation of hERG as a response to electrical pacing. Important future studies include determination of how electrical signals mediate hERG subunits 1a and 1b, where subunits play different regulatory roles but are both required for proper repolarization^48^,^49^,^50^. In addition, hERG has been shown to vary during heart failure and cardiac resynchronization therapy (CRT), clinical electrical pacing of the heart that can lead to left ventricular reverse remodelling and reduction of mortality^51^,^52^. hERG, downregulated during heart failure, can be restored by CRT^53^. While the precise link between electrical pacing *in vitro* and whole heart pacing remains unclear, our finding that hERG is upregulated following electrical stimulation, consistent with the upregulation of hERG following CRT, demonstrate the importance of electrical activity in modulating the function of ion channels. Future mechanistic studies into the role of electrical stimulation in modulating hERG may therefore yield insights relevant to clinical pacing.

Electrical stimulation also induced enrichment of cardiomyocytes expressing high levels of connexin and characterized by rapid depolarization. Cardiomyocytes with high amounts of connexin-40 and rapid depolarization were previously identified in developmental studies^34^,^54^,^55^, but the action potential morphology observed in this study is distinct from that in adult human Purkinje fibres, which have an even higher depolarization rate^56^,^57^. These cells may be rapidly conducting due to these properties. However, measurements of conduction velocities using either voltage-sensitive dyes or multi-electrode arrays give unreliable representations of the three-dimensional conduction within an EB^58^,^59^, or require the use of specially designed three-dimensional arrays^60^,^61^. Because electrical conditioning increased levels of Cx40 and Cx43 and caused greater resistance to electrical pacing stress, these cells may display greater electrical connectivity with the host myocardium and less susceptibility to electrical insults such as ventricular tachyarrhythmia^8^,^13^,^36^,^37^,^38^,^62^.

In summary, we found that electrical stimulation entrains the spontaneous frequency of human cardiomyocytes and provides conditioning cues for the generation of mature, structurally connected cells, therefore enabling future studies on the survival and engraftment of electrically preconditioned cardiomyocytes into the damaged myocardium, towards more effective stem cell therapy of the heart.

## Methods

### Derivation of human cardiomyocytes

Full sets of experiments were performed using hESC-derived cardiomyocytes (hES02 line). Sets of key studies were reproduced with iPS-derived cardiomyocytes (C2A line); these studies are clearly specified in the text.

hESCs and iPSCs were maintained and differentiated into cardiovascular lineages^26^,^27^. Briefly, hESCs and iPSCs were aggregated to form EBs in Ultra Low Cluster six well plates (Costar) for 24 h. Cardiac differentiation was performed in StemPro-34 media (Life Technologies) containing 1% L-glutamine (Sigma), 150 μg ml^−1^ transferrin (Roche Applied Science), 50 μg ml^−1^ L-ascorbic acid (Sigma), 4 × 10^−4^ M monothioglycerol (Sigma) and 1% penicillin/streptomycin (Sigma).

Cytokines were added to EB cultures of hESC and iPSC in a stage-specific manner. EBs were induced with 10 ng ml^−1^ BMP-4, 6 ng ml^−1^ Activin A and 5 ng ml^−1^ basic fibroblast growth factor (all from R&D) applied at days 1–4 of differentiation. At day 4, a Wnt inhibitor (150 ng ml^−1^ hDKK, R&D, and/or 1 μM XAV939, Tocris) was added for 4 days to specify cardiac mesoderm. From day 8, the cultures were maintained in media containing 10 ng ml^−1^ vascular endothelial growth factor and 5 ng ml^−1^ basic fibroblast growth factor (CM Media) until the end of the study, with media changes every 2–3 days.

Flow cytometry was performed on cells dissociated with TrypLE (Life Technologies) and fixed with 4% paraformaldehyde at day 5 for the expression of cardiac progenitors KDR and PDGFR-α (anti-KDR-phycoerythrin and anti-PDGFR-α-allophycocyanin, R&D) and at day 20 for the cardiomyocyte specific marker cTnT (clone 13-11, NeoMarkers)^27^. Cells were analysed on the LSRII flow cytometer and analysed using the FlowJo software. Cultures containing 50–60% cTnT-positive cells were typically obtained using this differentiation strategy and used for this study (Supplementary Fig. 1).

### Microbioreactor culture with electrical stimulation

Microbioreactors were fabricated, by moudling polydimethylsiloxane (Dow Corning) around a negative custom design^21^. Each bioreactor cassette had four rows of four wells, 5 × 5 mm in size. Carbon rods of 1.3 mm in diameter spanned the two ends of each row such that its four wells received the same stimulation. The bioreactor was sealed to a 75 × 25-mm glass slide (Fisher) using plasma treatment (PDC-002, Harrick Plasma).

Microbioreactors were loaded on day 20 with 10 differentiated EBs per well in CM Media and cultured for 3 days before electrical stimulation. Leads from an S88X Dual Output Square Pulse Stimulator (Grass Technologies) were connected to the carbon rods and the cells were stimulated continuously for 7 days, by square wave signals at 5 V cm^−1^ for 2 ms at 0.5, 1 and 2 Hz; the control group in the bioreactor did not receive stimulation. At the start of stimulation, EBs were assessed for capture to the provided electrical stimulus by visually observing that the stimulus rate matched exactly the rate of the EB. Cardiomyocytes fully captured the 2- and 1-Hz stimuli, but only partially captured the 0.5-Hz stimulus, as the faster native spontaneous beating rate overdrives the slower 0.5-Hz stimulus.

### Immunostaining

EBs were collected, dehydrated in 30% sucrose (Sigma) overnight and cryosectioned at 8 μm. Sections were thawed and rehydrated in calcium-free, magnesium-free PBS (Corning) for 5 min, permeabilized using 0.01% Triton-X (Sigma) and blocked with 10% horse serum (Sigma) in PBS for 20 min. Slides were stained with primary antibodies for 1 h using: mouse anti-Troponin-T (4 μg ml^−1^, Developmental Studies Hybridoma Bank, CT3), mouse anti-α-actinin (sarcomeric; 1:500 dilution, Sigma, A7811), mouse anti-connexin-43 (1:500, Abcam, ab11370), rabbit anti-hK_V_11.1, hERG (1:400 dilution, Alomone Labs, APC-062) or mouse anti-connexin-40 (1:100 dilution, Life Technologies, 36-4900). After three washes with PBS, slides were stained for 1 h with the following secondary antibodies: Goat Anti-Mouse IgG Alexa Fluor488 (2 μg ml^−1^, Life Technologies, A-11029) for Troponin-T, Goat Anti-Rabbit IgG Alexa Fluor594 (2 μg ml^−1^, Life Technologies, A11037) for Cx43, Cx40 and hERG, Goat Anti-Mouse IgG Alexa Fluor 647 (2 μg ml^−1^, Life Technologies, A-21236) for α-actinin. Slides stained for wheat germ agglutinin were preprocessed as above. Slides were incubated with 10 μg ml^−1^ Wheat Germ Agglutinin, Alexa Fluor594 Conjugate (Life Technologies) for 20 min and washed three times with PBS.

All slides were counterstained for 5 min with 4,6-diamidino-2-phenylindole (300 nM, Life Technologies). Fluorescence images were taken on the Olympus IX81 inverted microscope using the Hamamatsu C4742-95 camera.

### Transmission electron microscopy

Cells were fixed with 2.5% glutaraldehyde in 0.1 M Sorenson's buffer (PH 7.2) for 1 h. Cells are then postfixed with 1% OsO_4_ also in Sorenson's buffer for 1 h. After dehydration, cells were embedded in a mixture of Lx-112 (Ladd Research Industries, Inc.) and Embed-812 (EMS, Fortwashington, PA). Thin sections (60 nm) were cut on the MT-7000 ultramicrotome, stained with uranyl acetate and lead citrate, and examined on a JEOL JEM-1200 EXII electron microscope. Images were taken on an ORCA-HR digital camera (Hamamatsu) and recorded with an AMT Image Capture Engine. Sarcomere thickness was measured as the width of a single Z-line.

### Quantitative PCR

RNA was extracted with TRIzol reagent (Life Technologies) and quantified for yield and purity using NanoDrop (Thermo Scientific). Reverse transcription was performed using Ready-To-Go You-Prime First-Strand Beads (GE Healthcare Life Sciences) as per manufacturer protocol. Briefly, 2 μg of RNA was resuspended in 30 μl DEPC H_2_O (Life Technologies) with 0.3 μl of 0.2 μg μl^−1^ Random Primers (Life Technologies) and placed in a tube with the first-strand reaction mix. The mix was placed in a 37 °C water bath for 1 h. Quantitative PCR was performed on the StepOnePlus Real-Time PCR System (Applied Biosystems) where each well contained a reaction mix totalling 15 μl, with 5 μl of 1:10 diluted cDNA, 7.5 μl SYBR Green PCR Master Mix (Applied Biosystems) and 0.2 μM primers. The primers used are listed in Supplementary Table 1. The integrity of each primer was confirmed for the single product and each melting curve. The data were analysed using the ΔΔCt method, where samples were normalized to the calsequestrin expression and presented as an expression relative to the control group.

### Area change and frequency measurement

Videos of EBs were taken using an Olympus IX81 inverted microscope on a thermoregulated glass plate (Bioscience Tools) set to 37 °C. Phase-contrast videos were acquired using the Hamamatsu C4742-95 at 15 frames per second or with the AmScope MU500 at 30 frames per second. Video acquisition of EBs was taken at least 20 min after stopping electrical stimulation.

Acquired videos were analysed for frequency using a custom-made MATLAB-based GUI. Videos were analysed for area change using the same MATLAB-based GUI. Images of EBs were thresholded on a sliding scale, to isolate the EBs from the surrounding. Thresholding was applied to all frames within the video, and the area of each EB was measured for each time point. Data were normalized to the maximum area to calculate the fractional area change for each frame. Resulting graphs demonstrated peaks of maximum contraction, which were used to measure the frequency of contraction.

### Strain analysis

Videos were further analysed for strain generation based on previously described image correlation analysis^63^. Videos were first separated into individual frames using MATLAB and saved as a sequence of images that were loaded into PIVlab (v 1.3.2), a freely available particle-tracking tool built for MATLAB. The analysis window was masked to include only the EB of interest. As EBs beat, grey intensity patterns were measured by the built-in cross-correlation function comparing interrogation areas between two adjacent frames, and generated velocity vectors between the areas of similar intensity. Interrogation areas were made sufficiently small and were computed in three sequential passes (64 pixels, 32 pixels and 16 pixels) to obtain accurate correlations.

To generate heat maps of contraction velocity, data were set to the same colour limits (0–28 μm s^−1^). Strain rate was determined using built-in PIVlab functions, and compared with point-to-point contraction/relaxation data. An integral of the strain rate over the beating area was determined for each frame and averaged over the entire EB, resulting in a strain rate that could be traced over time. Positive strain rates for the contractile events were summed up to determine the cumulative strain, and divided by the number of contractions to determine the strain per beat.

### Calcium imaging

Cardiomyocytes were analysed for calcium transients after 7 days of stimulation. Cells were loaded with 5 μM Fluo-4 AM (Life Technologies) in Tyrodes Solution (140 mM NaCl, 5.4 mM KCl, 1 mM MgCl_2_, 10 mM glucose, 1.8 mM CaCl_2_ and 10 mM HEPES (pH 7.4 with NaOH; reagents from Sigma) containing 5 μM blebbistatin (Sigma) to reduce movement artefact for 15 min at 37 °C. The solution was then washed three times with Tyrodes Solution and imaged in Tyrodes containing 5 μM Blebbistatin. Videos were acquired with the Pike F-032 (Allied Vision Technologies) at 100 frames per second using SPLASSH software^64^.

Images were post-processed in MATLAB using a custom-made script. Briefly, a video was loaded into MATLAB and normalized against the minimum and maximum values captured in the whole video. Data were displayed for play back, to allow the user to choose a particular area of interest corresponding to an EB. Areas corresponding to background were also chosen. For each frame, the background fluorescence was subtracted from the mean fluorescent signal in the area of interest, giving a baseline-corrected fluorescence value. The signal was smoothed and plotted over time to determine calcium transients.

Three different metrics were derived from the calcium transient traces. The time to peak was measured as the time for the calcium signal to reach a maximum level for a particular beat at 10% intensity level above baseline. The decrease in calcium intensity from the peak intensity to the start of the next beat was fitted with an exponential curve, *Ae*^−*t*/*τ*^, where *τ* represented the time constant of decay. Full width half maximum, the width of the calcium transient at half of the maximum intensity, was determined as the amount of time elapsed from 50% of the maximum calcium signal during upstroke to the 50% of the maximum calcium signal during downstroke.

### Microarrays for gene expression

EBs were collected, snap frozen and used for RNA isolation and whole-genome expression profiling and analysis (by Expression Analysis, Durham, NC). The Illumina HumanHT-12 v4 Beat Chips were used to generate the expression profiles for the stimulation and control groups (*n*=3).

Each stimulation group was compared with the control using a permutation analysis for differential expression and tested for statistical significance. Genes with a frequency-dependent upregulation or downregulation of at least twofold were determined for all transcripts identified by the permutation analysis for differential expression analysis. Further identification of cardiac-specific genes was done manually. Heat maps were generated using GenePattern (Broad Institute)^65^, a free genomic analysis software package.

### Patch clamp for action potential recordings

After 7 days of stimulation, EBs were partially dissociated to create small cell clusters (3–6 cells) for patch-clamp studies. Cells were suspended in 75 μg ml^−1^ Liberase TM (Roche Applied Science) in calcium-free, magnesium-free Hank's Balanced Salt Solution (HBSS; Life Technologies) for 45 min at 37 °C. Dissociated cells were resuspended in CM media and plated onto 1:20 diluted Matrigel, growth factor-reduced (BD)-coated glass coverslips. Cells were acclimated to the glass coverslips for 3 days without any additional electrical stimulation before patch clamping.

For AP recordings, the coverslips with dissociated cells were put into the experimental bath and superfused with solution of the following composition (in mM): 140 NaCl, 5.4 KCl, 1.0 CaCl_2_, 1.0 MgCl_2_, 5.0 HEPES and 10 dextrose (pH 7.4, 35 °C). Perforated patch-clamp recordings used a computer equipped with pCLAMP 8 software, a Digidata 1322 A series interface, and Axopatch 1C amplifier (Molecular Devices, Sunnyvale CA). Borosilicate glass pipettes (2–3 MΩ, Sutter Instrument, Novato CA) contained (in mM) 130 aspartic acid, 146 KOH, 10 NaCl, 2.0 CaCl_2_, 1.0 MgCl_2_, 5.0 EGTA, 10 HEPES and 2.0 MgATP (pH 7.2). Water-soluble amphotericin B was added to the pipette solution to reach final concentration of 0.5 mg ml^−1^. Superfusion was provided by two lines, one containing a control solution and one containing 5 μM E-4031, embedded into the heating unit with a tip distanced at 0.5 mm from the small cell cluster.

Regularly beating cell clusters of small size (86.7±3.4 μm in diameter, *n*=65) were used for patching. After patching, the capacitance currents were recorded in voltage clamp mode to monitor pore formation within the patch. Within 3–4 min, an amphotericin-induced increase of electrical access was detected, at which point current clamp mode was used to monitor spontaneous APs. Recordings were made after the peak amplitude and MDP reached stable levels.

To analyse results, Clampfit, version 9, was used. Peak amplitude, MDP, maximal upstroke velocity (d*V* d*t*_max_^−1^) and rate of spontaneous APs were measured automatically with the Clampfit Event Detection option. The take-off potential and AP duration at 30, 50 and 90% repolarization from peak amplitude (APD30, APD50 and APD90) were also found using the analysis option or by individual measurements. To determine whether a particular cell qualified as a ‘rapid depolarizer,' all of the d*V* d*t*_max_^−1^ data were examined and whether the d*V* d*t*_max_^−1^ was an outlier. The cutoff for an outlier was defined as Q3+1.5 × interquartile range (quartile 3+1.5 times the interquartile range)^66^,^67^,^68^.

### Drug studies

To determine the effect of E-4031 (an inhibitor of the *KCNH2* channel) on beating frequency, videos were taken at the baseline (drug-free culture medium), with the drug, and following washout of the drug. Baseline videos were first acquired for EBs from all groups, in fresh CM Media after equilibration. After baseline videos were acquired, 5 μM of E-4031 (Tocris) were supplemented to fresh CM Media and the EBs were allowed to equilibrate for 20 min before taking videos. EBs were then washed with CM media three times, allowed to equilibrate for 20 min, and videos of the same EBs were taken the third time to determine the frequency of beating after the drug washout.

To determine the effect of constitutive culture with an inhibitor of *KCNH2*, 5 μM E-4031 was added to the media at the start of the stimulation period. Videos were taken on days 1, 3 and 7 following 20 min of equilibration to fresh media containing E-4031. At the end of the culture period, the cardiomyocytes were washed with fresh CM Media without E-4031 three times, allowed to equilibrate for 20 min and re-evaluated for frequency.

To examine the effects of isoproterenol on cardiomyocytes, videos were first taken at the baseline in fresh CM media after equilibration, and then in CM media containing 1 μM isoproterenol (Sigma).

### Formation of chimeric EBs

EBs were dissociated with 75 μg ml^−1^ Liberase TM (Roche Applied Science) in calcium-free, magnesium-free HBSS(Life Technologies) for 45 min at 37 °C. Cells were centrifuged and washed with HBSS twice, mixed at specified ratios and reaggregated in Ultra Low Cluster 24-well plates (Corning) for 3 days. Videos of spontaneous beating were then acquired.

To validate that resultant EBs contained a mix of both cell types, one population of EBs were dyed with CellTracker Red CMTPX Dye (Life Technologies) and the other half with CellTracker Green CMFDA Dye (Life Technologies) before cell dissociation and reaggregation.

### Measurement of apoptosis

Following 7 days of electrical stimulation, EBs were subjected to an additional 24 h of stimulation at 3 Hz, 5 V cm^−1^, 2 ms pulse duration using the S88X Dual Output Square Pulse Stimulator (Grass Technologies). Cardiomyocytes were collected and sectioned as described above. Apoptosis was assayed using the Click-iT TUNEL Alexa Fluor594 Imaging Assay (Life Technologies) according to the manufacturer protocol. Slides were counterstained with 4,6-diamidino-2-phenylindole and imaged using the Hamamatsu camera C4742-95 as described above.

### Statistical analysis

Quantitative PCR results were presented as a fold change relative to the control, and analysed using one-way analysis of variance (ANOVA; *n*≥3, *P*<0.05). Video data were presented as the average±s.e.m. of the fold change, and the differences between experimental groups and time points were analysed using two-way ANOVA (*n*≥30, *P*<0.05). Calcium transient metrics were presented as average±s.e.m. and differences between experimental groups were determined using one-way ANOVA (*n*≥23, *P*<0.05). Contractile analysis was presented as average±s.e.m. of the strain, and analysed using one-way ANOVA (*n*=3, *P*<0.05). Effects of inhibitor addition and washout on beating frequency were analysed using two-way ANOVA (*n*=3, *P*<0.05). Parameters of action potential measurements were shown as average±s.e.m. and the significance was determined by two-way ANOVA (*n*≥4, *P*<0.05). All ANOVA tests were subsequently analysed using Tukey's *post hoc* test.

## Additional information

**Accession codes:** Microarray data have been deposited in the Gene Expression Omnibus (GEO) under the accession code GSE74669.

**How to cite this article:** Eng, G. *et al.* Autonomous beating rate adaptation in human stem cell-derived cardiomyocytes. *Nat. Commun.* 7:10312 doi: 10.1038/ncomms10312 (2016).

## Supplementary Material

Supplementary Information Supplementary Figures 1-13 and Supplementary Table 1

Supplementary Movie 1 Representative spontaneously contracting unstimulated embryoid body.

Supplementary Movie 2 Representative spontaneously contracting 0.5 Hz stimulated embryoid body.

Supplementary Movie 3 Representative spontaneously contracting 1 Hz stimulated embryoid body.

Supplementary Movie 4 Representative spontaneously contracting 2 Hz stimulated embryoid body.
