# Supplementary material for: Autonomous beating rate adaptation in human stem cell-derived cardiomyocytes
Source: Nat Commun. 2016 Jan 19;7:10312. doi: 10.1038/ncomms10312 (PMC4735644; doi:10.1038/ncomms10312)
Supplement: Supplementary Information — Supplementary Figures 1-13 and Supplementary Table 1 [file ncomms10312-s1.pdf]

Supplementary Information:

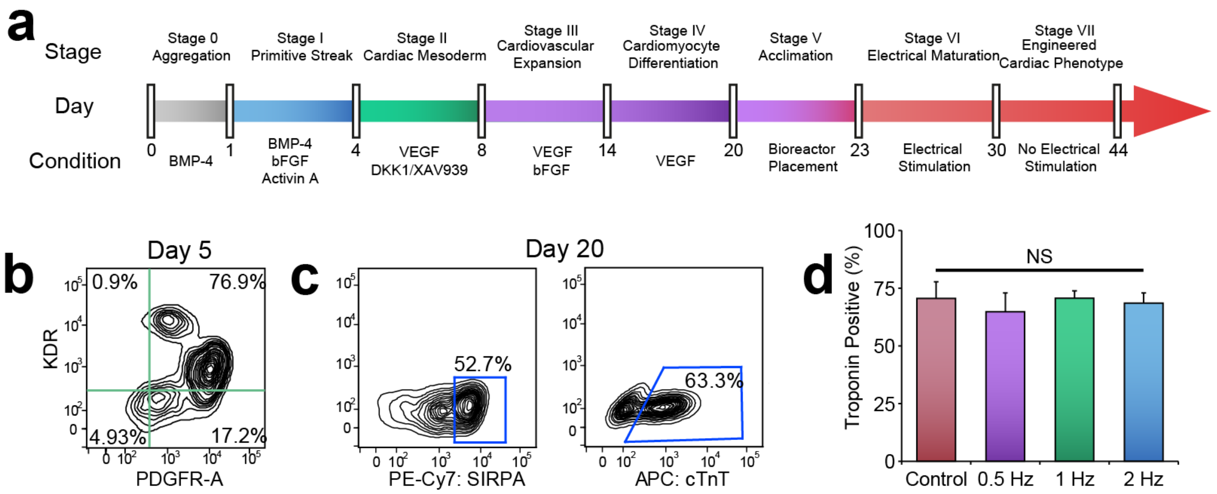

Supplementary Figure 1 | Cardiomyocyte differentiation. (**a**) Staged differentiation procedure used to generate stem cell-derived cardiomyocytes. (**b,c**) Flow cytometry of cardiomyocytes at two time points (early and late) during the differentiation. (**b**) Contour plot showing staining for cardiac progenitors PDGFR-A and KDR at day 5. (**c**) Contour plots for mature cardiac markers SIRPA and cTnT, showing >50% positively staining cells at day 20. (**d**) Percent of troponin-positive cells in embryoid bodies by immunofluorescence staining.

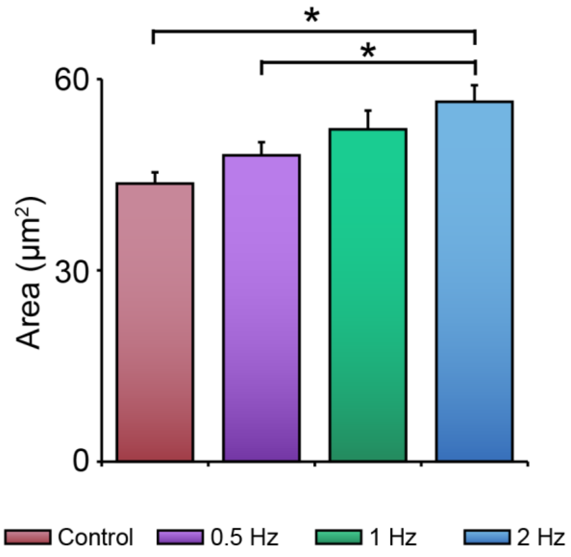

Supplementary Figure 2 | Effect of Electrical Stimulation on Cardiomyocyte Hypertrophy. Wheat germ agglutinin quantification of cardiomyocytes in different stimulation groups. Cell borders were traced to give cell size (Average cell area ±s.e.m). *Statistically significant differences between groups (n>100, $p<0.05$, one-way ANOVA with post hoc Tukey test).

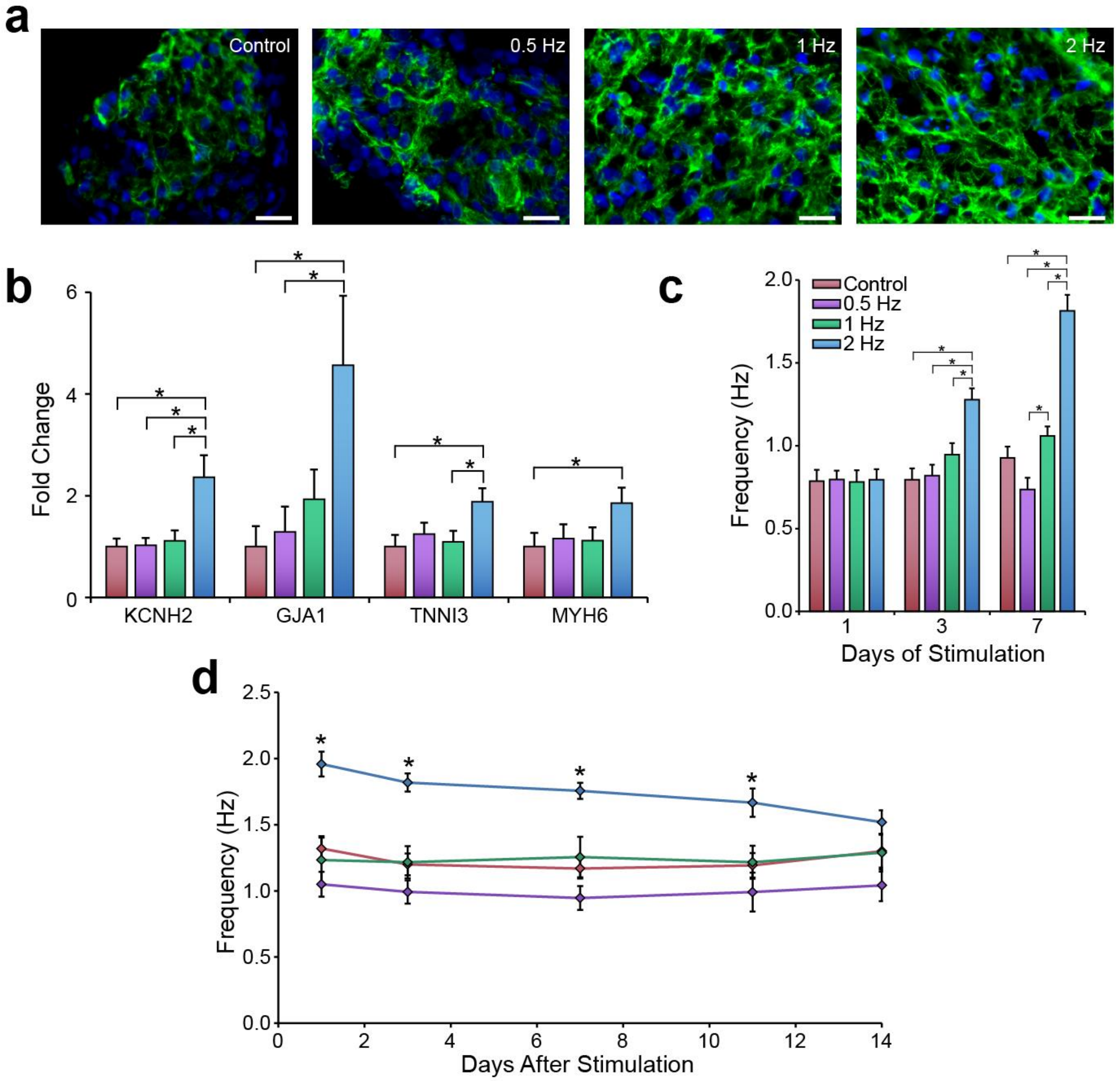

Supplementary Figure 3 | Effects of electrical stimulation on iPS cell derived cardiomyocytes. (**a**) Representative immunostains of troponin (green) and DAPI (blue). Scale = 50 µm (**b**) Quantitative PCR of cardiac markers (n=13, p<0.05). (**c**). Frequency of autonomously beating cardiomyocytes ± s.e.m. as a function of days of stimulation (n=15, p<0.05). (**d**). Differential beating frequency was maintained for approximately two weeks following the end of the seven day stimulation period (n=10, p<0.05). All statistical testing was done using one-way ANOVA with post hoc Tukey tests.

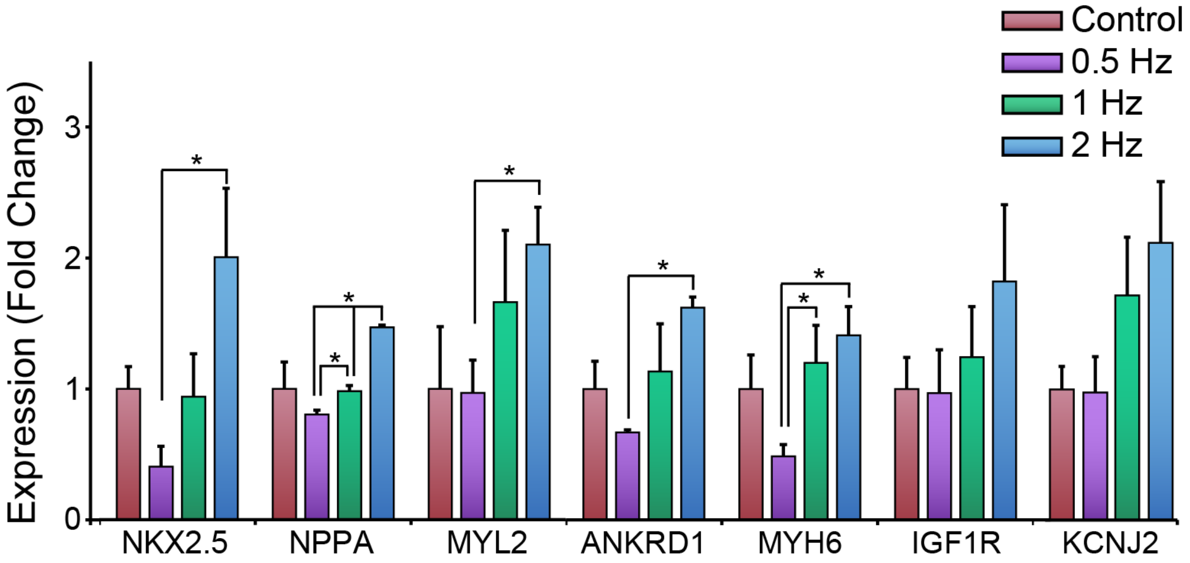

Supplementary Figure 4 | Quantitative PCR of cardiac markers. Data are shown as Average ±s.e.m. of the fold change relative to unstimulated controls. *Statistically significant differences between groups; connected lines denote differences between the individual groups and the 2 Hz group (n=3, $p<0.05$, one way ANOVA with post hoc Tukey test).

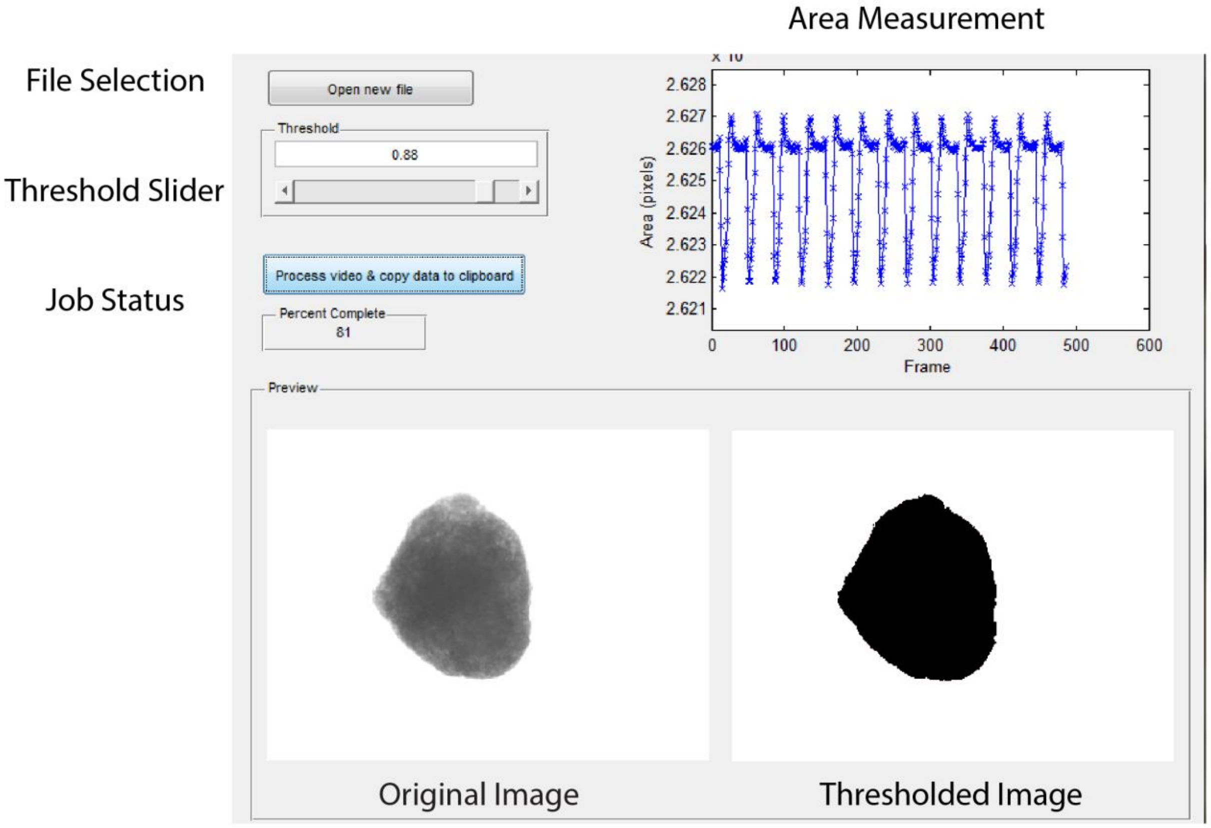

Supplementary Figure 5 | MATLAB graphical user interface for determining fractional area change in contracting EBs. The user chose a video file of interest, adjusted the threshold of the image sequence using a sliding scale, and conducts the area measurements in real time.

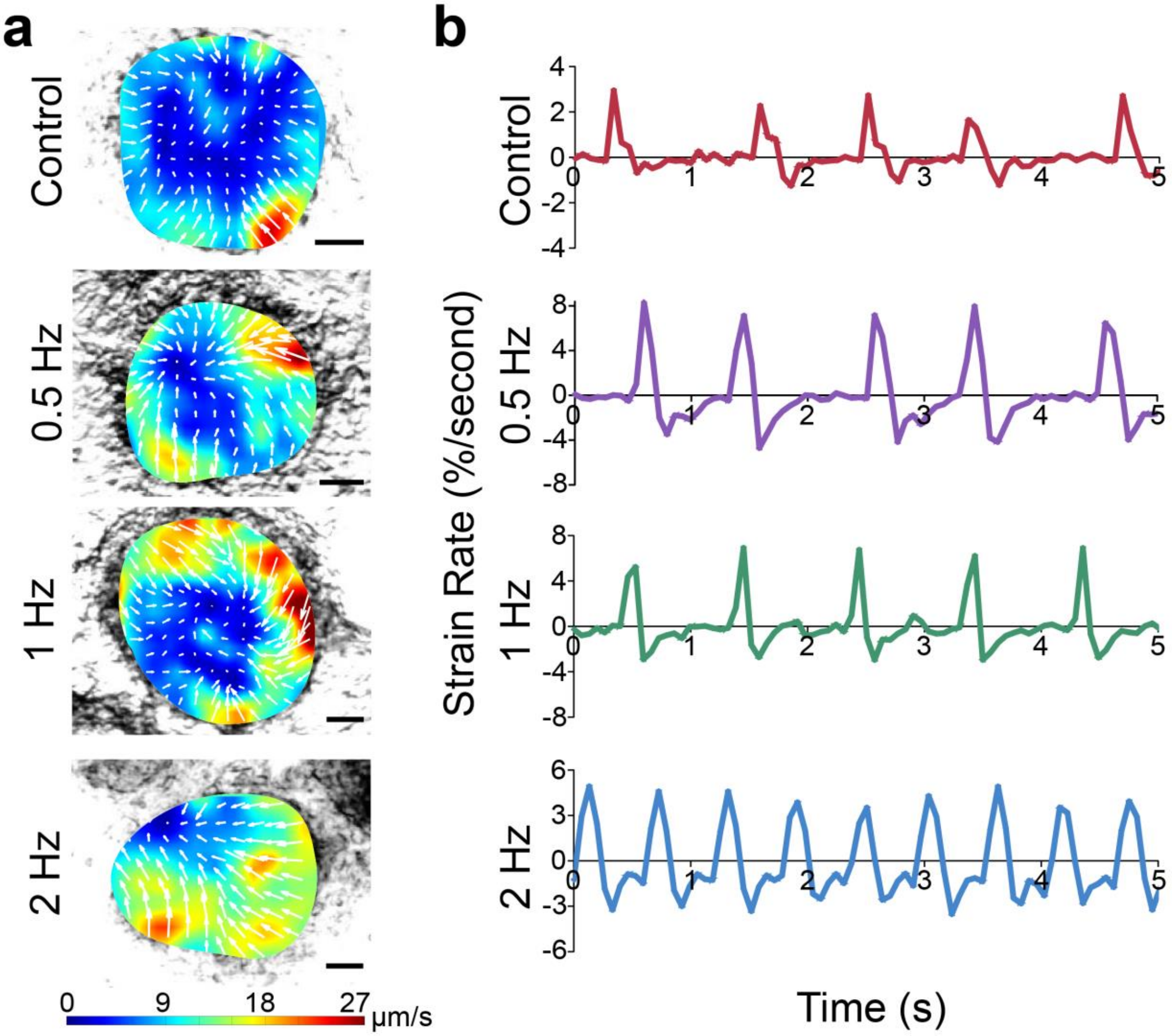

Supplementary Figure 6 | Strain analysis. (**a**) Sample velocity maps of contracting EBs. Red colors and longer arrows represent highest velocities, and blue colors and shorter arrows represent lowest velocities. Scale bar = 50 μm. (**b**) Representative overall strain of an EB over time. Positive strains refer to contraction while negative strains refer to relaxation.

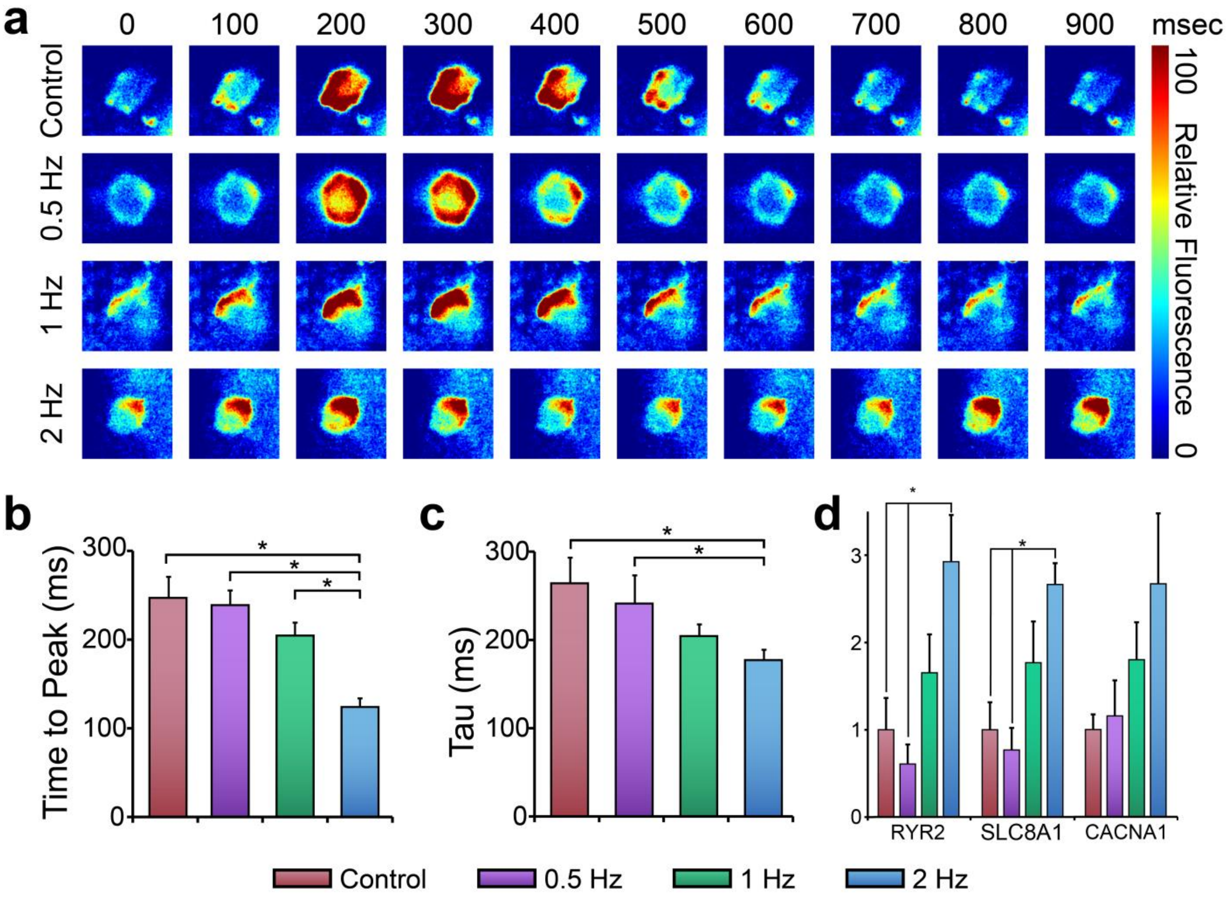

Supplementary Figure 7 | Calcium handling. (**a**) Representative calcium heat-maps recorded over a 1-s period; red denotes highest relative fluorescence and blue denotes lowest relative fluorescence. (**b**) Time to peak and (**c**) The calcium decay rate constant, that was smaller in stimulated groups compared to unstimulated controls. * Significant difference between groups (n=23, $p<0.05$). (**d**) Quantitative PCR of calcium handling genes, displayed as Average ± s.e.m. of the fold change relative to unstimulated control; lines denote differences between multiple groups and the 2 Hz group (n=3, $p<0.05$). All statistical testing was done using one-way ANOVA with post hoc Tukey test

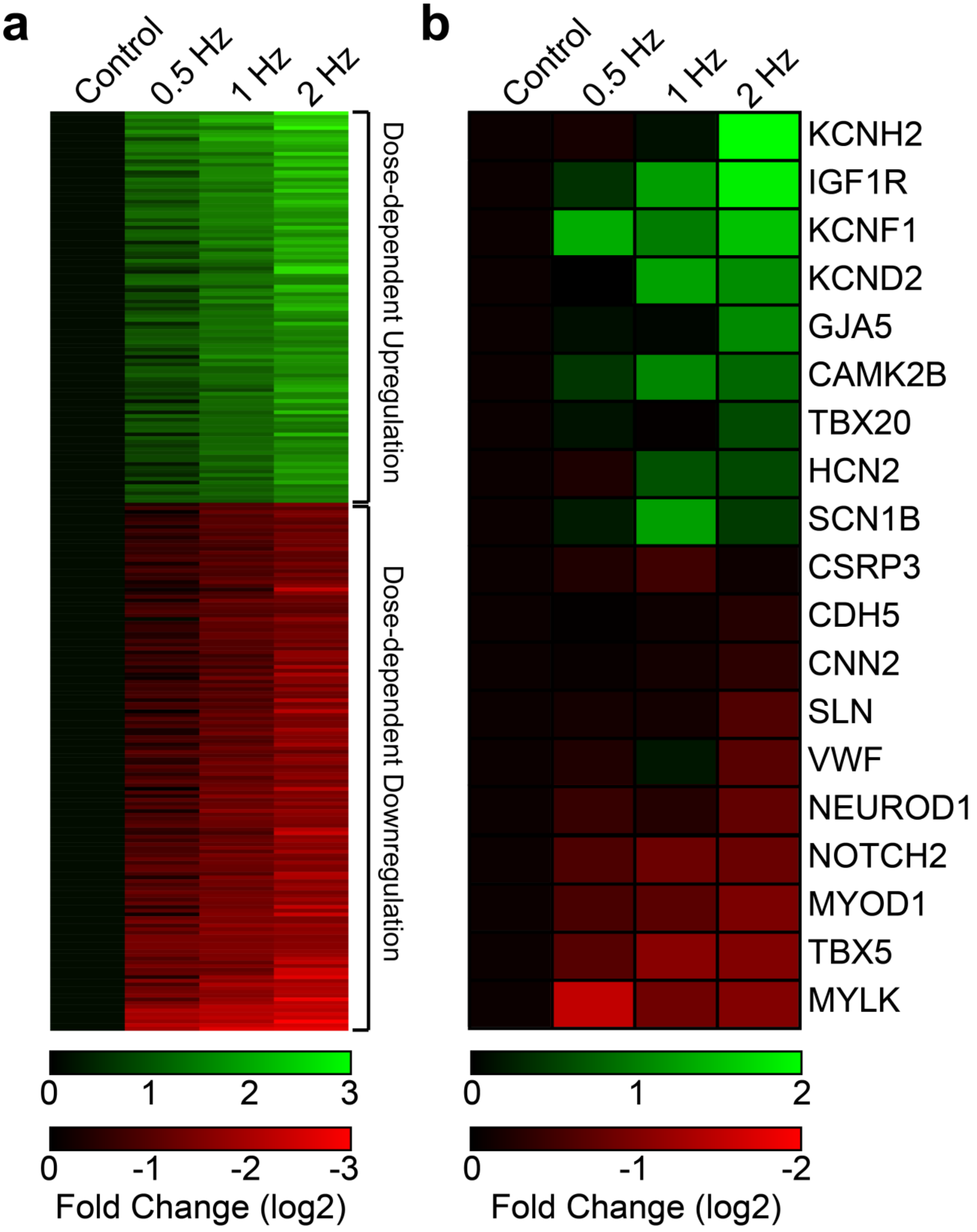

Supplementary Figure 8 | Gene expression microarrays. (**a**) Genome wide profile of all genes upregulated or downregulated with stimulation of hESC-CMs. A total of 106 genes were upregulated and 143 genes were downregulated at least 2-fold in a frequency-dependent manner. (**b**) A subset of salient genes upregulated or downregulated following electrical stimulation.

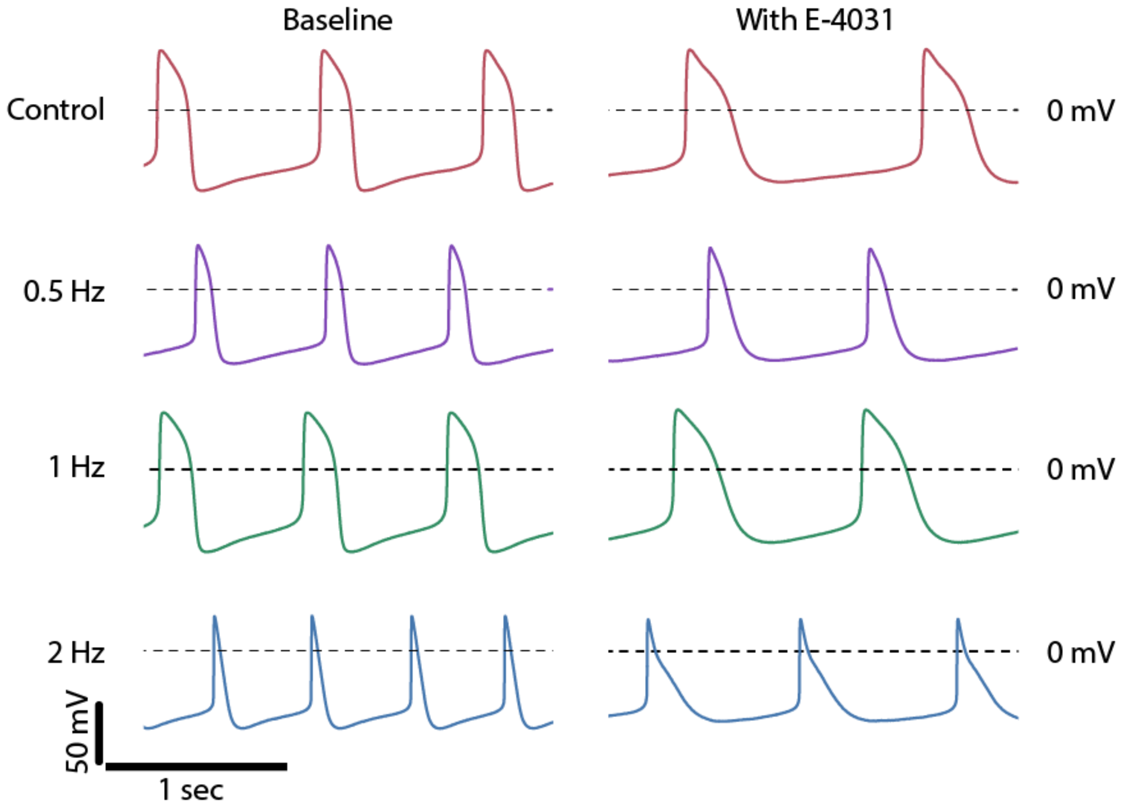

Supplementary Figure 9 | Action Potentials. Representative action potentials are shown for the four stimulation groups, at baseline and after the application of hERG antagonist E-4031.

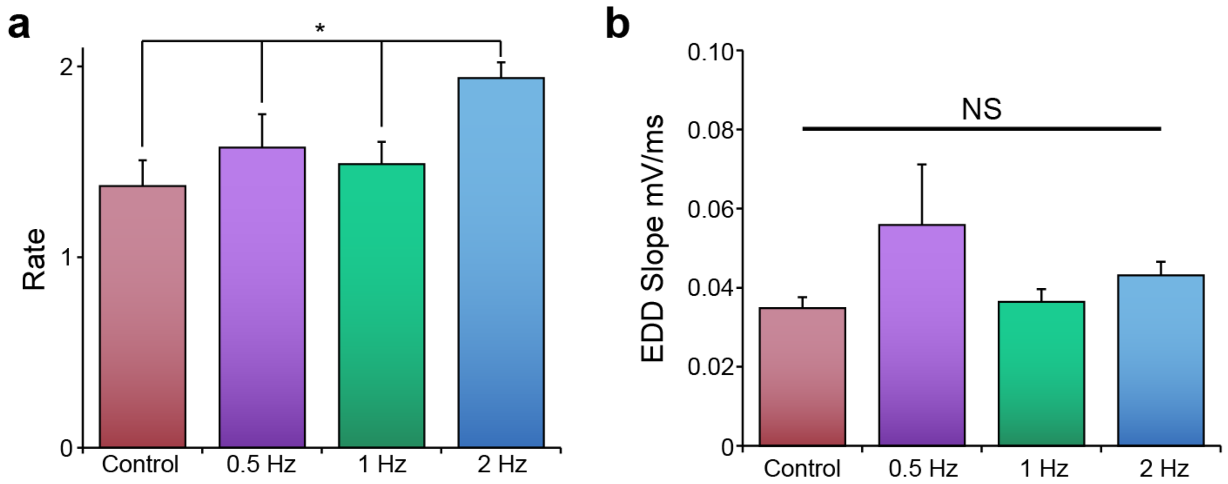

Supplementary Figure 10 | Action Potential Data. (**a**) Rate of dissociated cardiomyocytes matched the adapted rates of the embryoid bodies. (**b**) End-diastolic depolarization slope was not significantly different between groups. * Significant difference between 2 Hz electrically stimulated groups and the other three groups. NS denotes non-significance between groups (Control: n=13; 0.5 Hz: n=5; 1 Hz: n=9; 2 Hz: n=15; $p<0.05$, one way ANOVA with post hoc Tukey test).

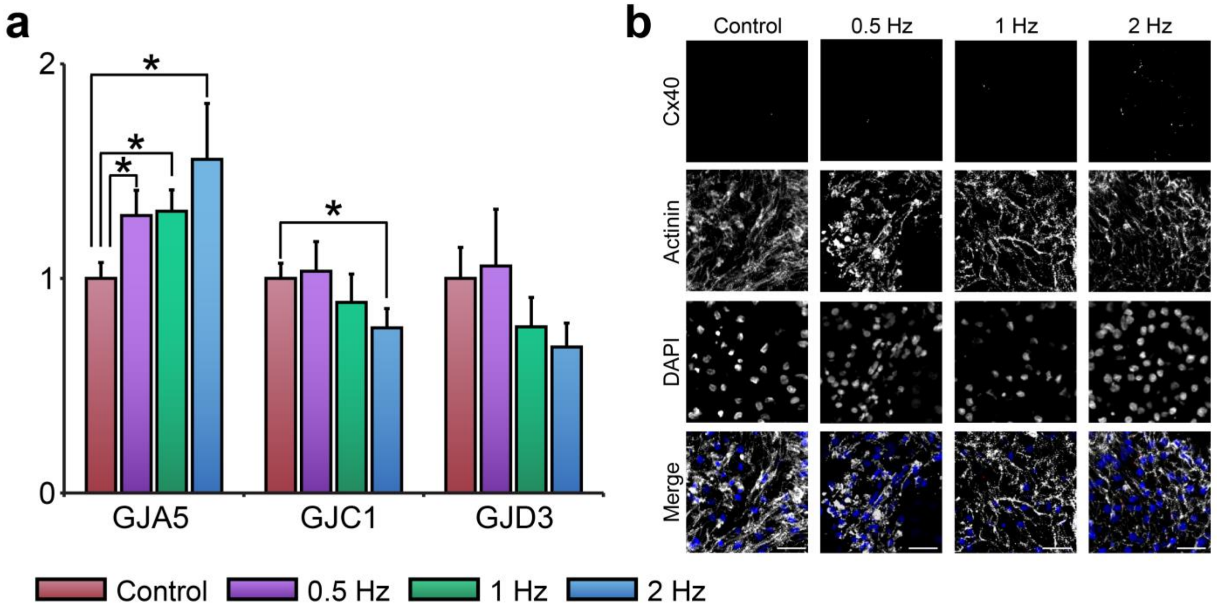

Supplementary Figure 11 | Expression and presence of Connexin-40. (**a**) Quantitative PCR of gap junctions, where expression is presented as Average ±s.e.m. of the fold change relative to control. *Statistically significant differences between groups; connected lines denote significant differences between the groups and the 2 Hz group (n=9, $p<0.05$, one way ANOVA with post hoc Tukey test). (**b**). Immunostains demonstrating increasing levels of Cx40 (red staining) with increasing frequency of stimulation. Slides were counterstained with actinin (gray) and DAPI (blue). Scale = 25 µm

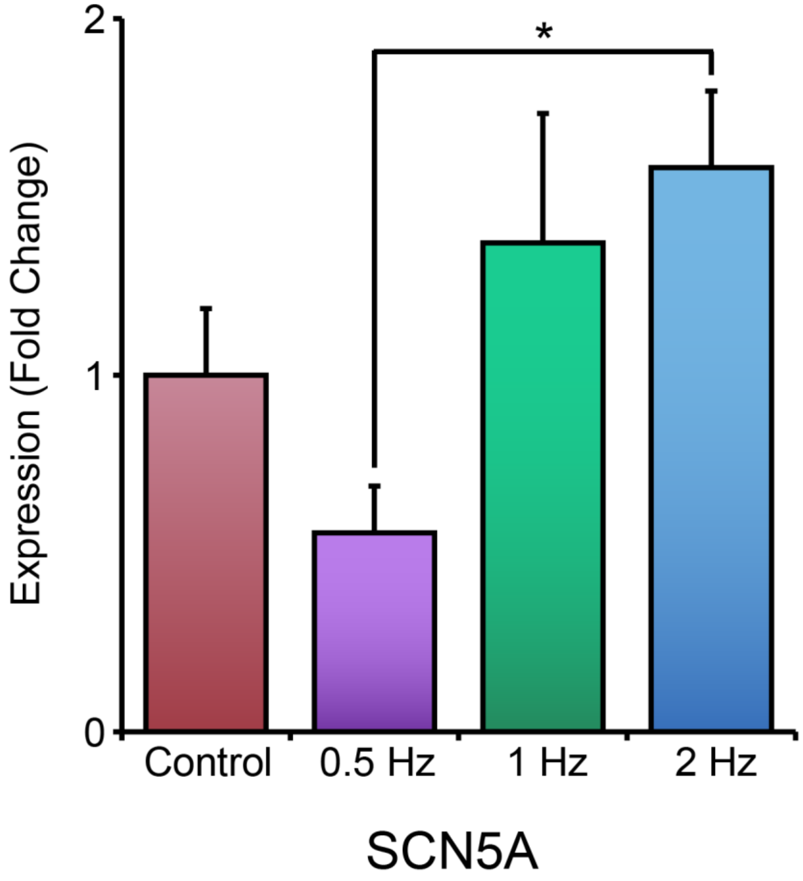

Supplementary Figure 12 | Quantitative PCR of *SCN5A*. Data are shown as Average ± s.e.m. of the fold change relative to unstimulated controls. *Statistically significant differences between groups (n=3, $p<0.05$, one way ANOVA with post hoc Tukey test).

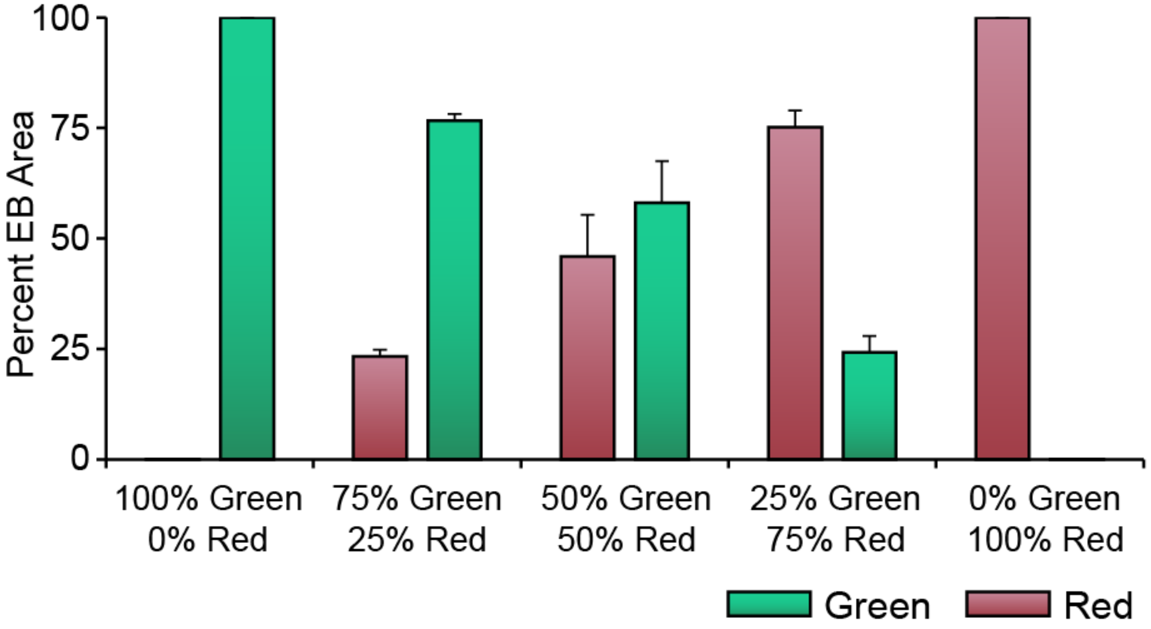

Supplementary Figure 13 | Chimeric Embryoid Body Formation. Cardiomyocytes stained fluorescent red or green were dissociated and reaggregated for three days at different ratios. Percent EB area displaying each fluorescence is shown (average ±s.e.m.; n=5).

	Forward	Reverse
GAPDH	GGAGCGAGATCCCTCCAAAAT	GGCTGTTGTCATACTTCTCATGG
CASQ2	GGCAGAAGAGGGGCTTAATTT	GAAGACACCGGCTCATGGTAG
TNNI3	TTTGACCTTCGAGGCAAGTTT	TGCAGAGATCCTCACTCTCCG
GJA1	GGGACAGCGGTTGAGTCAG	TGTTACAACGAAAGGCAGACTG
TTN	CCCCATCGCCCATAAGACAC	CCACGTAGCCCTCTTGCTTC
Nkx2.5	CCTCAACAGCTCCCTGACTC	CTCATTGCACGCTGCATAAT
NPPA	CAACGCAGACCTGATGGATTT	AGCCCCCGCTTCTTCATTC
MYL2	TTGGGCGAGTGAACGTGAAAA	CCGAACGTAATCAGCCTTCAG
ANKRD1	CGTGGAGGAAACCTGGATGTT	TCTCGGGCGCTAATTTTTGCT
MYH6	GCCCTTTGACATTCGCACTG	CGGGACAAAATCTTGGCTTTGA
IGF1R	AAAATGCTGACCTCTGTTACCTC	GGCTTATTCCCCACAATGTAGTT
RYR2	ACAACAGAAGCTATGCTTGGC	GAGGAGTGTTCGATGACCACC
SLC8A1	ACAACATGCGGCGATTAAGTC	GCTCTAGCAATTTTGTCCCCA
CACNA1	AATCGCCTATGGACTCCTCTT	GCGCCTTCACATCAAATCCG
KCNH2	CAACCTGGGCGACCAGATAG	GGTGTTGGGAGAGACGTTGC
GJA5	AGAGTGTGAAGAAGCCCACG	AACAGATGCCAAAACTTCTGCT
GJC1	AGCTGTAGGAGGAGAATCCATC	TGCAAACGCATCATAACAGACA
GJD3	GCTGTTCGTCGTCTACTCCAT	ACCGCGAAATAGAAGAGCACG

Supplementary Table 1 | Primers used for qPCR
